# The Medical Society of the County of Erie

**Published:** 1897-02

**Authors:** 


The report was accepted and the committee discharged.

The officers elected for the ensuing year are: President, Dr. E. B. Angell, Rochester; first vice-president, Dr. Wm. C. Krauss, Buffalo; second vice-president, Dr. F. H. Stephenson, Syracuse. The secretary and the treasurer continue in office, their terms not having expired.

Upon motion, a vote of thanks was extended to the president and the secretary for their efforts in making the meeting a successful one.

---

### THE MEDICAL SOCIETY OF THE COUNTY OF ERIE.

THE Medical Society of the County of Erie held its seventy-sixth annual meeting, January 12, 1896, in the rooms of the Buffalo Academy of Medicine.

The president, Dr. J. G. Thompson, of Angola, took the chair and opened the proceedings promptly at 10 o'clock. After the minutes of the semi-annual meeting had been read and approved, Dr. John Cronyn asked leave to correct an inaccuracy which appeared in Dr. Edward Storck's paper, as published with the annual report of the proceedings of the seventy-fifth anniversary of the society, in relation to the organisation of the medical department of Niagara University. This was granted.

Dr. G. W. McPherson, chairman of the committee on membership, presented the names of the following applicants and recommended their admission to the society: Drs. Jane N. Frear, Frederick W. Hayes, Edward E. Koehler, Earl P. Lothrop, E. T. Rulison, A. E. Woehnert, Marion Marsh, Cora Billings Lattin and Henry W. Lattin. They were unanimously elected and subsequently those present were introduced.

Applications for membership were received from the following: Drs. Lorenzo Burrows, Jr., Samuel Outwater, Mary M. Huntley, J. Glen Ernest, F. W. McGuire and Felix Hintz. Referred to the committee on membership.

The president named as a committee on nominations, Drs. Wm. C. Krauss, G. W. McPherson, J. J. Walsh, W. M. Ward and E. Wende.

Dr. Edward Clark's report as treasurer was referred to an auditing committee, consisting of Drs. Wm. C. Phelps and Henry Lapp.

Dr. Ernest Wende, of the committee on a permanent home for the society, reported encouraging progress. The report was accepted and the committee continued. He also reported, at the request of the president, on the work done by the medical committee for the national G. A. R. encampment, to be held in Buffalo during the summer of 1897, and read a list of committee appointments.

Dr. Coakley, chairman of the board of censors, made a detailed verbal report on the work performed by the censors during the past year. He stated that no formal report had been prepared, as no violations of the state medical law had been referred to them for investigation. A number of cases, however, had been looked after. The first was referred to them by Dr. Edward Storck, who enclosed a circular of an alleged illegal practitioner. This man was found, however, to be a graduate of Bellevue Hospital some years ago, and to have registered in the county clerk's office in 1880. Another case from Brant, referred to the board by the president, was investigated, but the offending party suddenly disappeared. Two men on Broadway, who were charged with illegal practice, were found to be simply selling medicine. Another young man, who was charged with illegally practising, was found to be a graduate of Buffalo University and otherwise legally qualified. The report was accepted.

Dr. BENEDICT said that under present restrictions less harm resulted from illegal practitioners in medicine than from those practising in a legal manner, but in violation of medical ethics.

Dr. COAKLEY said they could only reach such as are members of this society. An effort had been made to secure an amendment to the medical law, which would remedy this evil, but it was found that public opinion was not yet ripe for it.

The auditing committee reported having found the treasurer's accounts correct and, on motion, their report was accepted and the committee discharged.

On motion of Dr. KRAUSS, the usual sum of $50 each was allowed to the secretary and treasurer for services.

Dr. LUCIEN HOWE offered the following resolution, which was adopted :

WHEREAS, The disease known as purulent ophthalmia of infancy produces about one-fifth of all the blind in asylums, and also more blind than any other disease, and

WHEREAS, The procedure known as Credé's method, is not only the most efficient means for preventing this disease, but when properly employed, reduces very greatly the percentage of such cases, therefore,

*Resolved*, That in the opinion of this society, Credé's method for the prevention of purulent ophthalmia, should be made obligatory in all public institutions.

The committee on nominations made the following report : For president, Dr. Henry R. Hopkins ; for vice-president, Dr. Hiram P. Trull, of Williamsville ; for secretary, Dr. Franklin C. Gram ; for treasurer, Dr. Edward Clark ; for librarian, Wm. C. Callanan ; for censors, Drs. John B. Coakley, C. E. Congdon, T. F. Dwyer, Irving W. Potter, Gustave A. Pohl ; for committee on membership, Drs. G. W. McPherson, John J. Walsh and W. W. Potter.

Dr. W. W. POTTER moved that the secretary cast the ballot of the society for Dr. Hopkins for president.

Dr. HOPKINS stated that he appreciated the great kindness and honor, but it would be impossible for him to accept and fulfil the requirements of the office. The society, however, entertained Dr. Potter's motion, which was carried amid applause.

A similar motion prevailed for the office of vice-president and the remainder of the ticket, when all the nominees were declared duly elected.

The committee appointed to look into the County Hospital matter had no report to make and was discharged.

President Thompson then called the vice-president to the chair and delivered his annual address on Surgery of the war of 1861-65.

On motion of Dr. Cronyn, a vote of thanks was given the retiring president for his address, as well as for the faithful performance of his duties, and a copy was likewise requested for publication.

On motion of Dr. Coakley that portion of the president's address requiring the action of the Society was referred to a special committee consisting of Drs. W. W. Potter, E. L. Bissell, W. H. Gail, D. W. Harrington, U. C. Lynde, T. M. Johnson and J. G. Thompson.

Dr. F. W. Hinkel read a paper on Earache, its significance and management; Dr. P. W. Van Peyma one on Albuminuria of pregnancy, and Dr. T. B. Carpenter one on Genito-urinary epithelium.

A vote of thanks was extended to the essayists and copies of their papers requested for publication in the Buffalo Medical Journal.

Dr. Krauss stated that the Medical Society of Central New York would meet in Buffalo next October. On his motion an appropriation of $50 was made, with which to entertain the delegates at luncheon, and a committee appointed with power.

The president appointed as such committee Drs. Wm. C. Krauss, F. C. Gram and Edward Clark.

The society adjourned at 1 p. m.

---

Oxygen in Diabetes.—To a man sixty years of age Ascoli (*Il Policlinico*) gave large quantities of oxygen each day for a little over three months. During this time the quantity of urine diminished, its specific gravity decreased, sugar was reduced to a small proportion and in two months entirely disappeared; three months later the sugar had not reappeared, although the patient had gone back to a diet of starchy foods. He also gained materially in weight.—*Med. Age.*

